# ﻿Four new *Planothidium* species (Achnanthidiaceae, Bacillariophyceae) from the Karst region of Guizhou in China

**DOI:** 10.3897/phytokeys.259.150757

**Published:** 2025-06-25

**Authors:** Yun Li, Lin-Xin Lu, Hui-Wen Zhou, Pan Yu, John Patrick Kociolek, Wan-Ting Pang, Quan-Xi Wang, Qing-Min You

**Affiliations:** 1 College of Life Sciences, Shanghai Normal University, Shanghai 200234, China; 2 School of Environment and Geographical Sciences, Shanghai Normal University, Shanghai 200234, China; 3 Museum of Natural History and Department of Ecology and Evolutionary Biology, University of Colorado, UCB 218, Boulder CO80309, USA

**Keywords:** Diatoms, karst, monoraphid, new taxa, *Planothidium*, taxonomy

## Abstract

Four new species of monoraphid diatoms belonging to the genus *Planothidium* were studied and described from the karst region of Guizhou Province, China. The morphological characteristics of these new species were observed and documented using light and scanning electron microscopy. *Planothidiumpseudoreichardtii***sp. nov.** exhibits a broad sinus at the central area on the interior of the rapheless valve, linear shallow depressions and a unique enlarged areola at the end of the striae on the exterior rapheless valve. *Planothidiumliboensis***sp. nov.** is characterized by 4–5 rows striae on both valves, a cavum at the central area on the interior rapheless valve and irregular depressions on the exterior rapheless valve. *Planothidiumangustirostratum***sp. nov.** features elliptical valves with narrowly rostrate apices, a cavum at the central area of interior rapheless valve and circular depressions on the exterior rapheless valve. *Planothidiummaolanensis***sp. nov.** has elliptical valves without protracted apices, coarsely-spaced striae on the one side of the central area of the both valves. These four new species were compared with other similar *Planothidium* taxa. In addition, ecological information was provided and the stability of some features was discussed.

## ﻿Introduction

Monoraphid diatoms are particularly intriguing due to their structural asymmetry, characterized by the presence of a raphe on one valve and its absence on the other (Kulikovskiy et al. 2016). The genus *Planothidium* Round & Bukhtiyarova was established to accommodate monoraphid diatoms previously classified within the genus *Achnanthidium* Kützing (Round and Bukhtiyarova 1996). Currently, approximately 129 accepted taxa of *Planothidium* are recorded in AlgaeBase (Guiry and Guiry 2024). The genus is characterized by the presence of multiseriate striae on both the raphe valve (RV) and rapheless valve (SV), as well as asymmetrical structures in the central area of the rapheless valve in many species (Morales 2006). Since its establishment, *Planothidium* has undergone significant taxonomic refinement, with studies describing new species and exploring its morphological and ecological diversity. The application of scanning electron microscopy and molecular techniques has further enhanced the differentiation of *Planothidium* species and deepened our understanding of their morphological features (Novis et al. 2012; Álvarez-Blanco and Blanco 2013; Van De Vijver et al. 2013; Jahn et al. 2017; Van De Vijver and Bosak 2019; Wetzel et al. 2019).

Morphological studies of *Planothidium* have focused on key characteristics, such as the structure of the rapheless valve’s central area, valve outline, and striae patterns. Morales (2006) proposed a classification framework for *Planothidium* species based on the central area morphology of the rapheless valve, dividing them into four groups: (1) species with continuous, uninterrupted striae in the central area, such as *P.daui* (Foged) Lange-Bertalot; (2) species with a variable, clear space in the central area that does not show any indentation or convexity, such as *P.minutissimum* (Krasske) Lange-Bertalot; (3) species with a depressed central area without striae, such as *P.lanceolatum* (Brébisson ex Kützing) Lange-Bertalot; (4) species with a depressed region on one side of the central area, which is capped to form a cave-like structure called a “cavum”, such as *P.biporomum* (Hohn & Hellerman) Lange-Bertalot (Morales 2006). In the study by Stancheva et al. (2020), *Planothidium* species were categorized into three groups: those with a sinus (single horse-shoe shaped mark), such as *P.lanceolatum*; those with a cavum (double horse-shoe shaped mark), such as *P.frequentissimum* (Lange-Bertalot) Lange-Bertalot; and those lacking both a sinus and a cavum, such as *P.minutissimum* (Krasske) Morales and *P.daui* (Foged) Lange-Bertalot. Moreover, beyond classic morphological measurements (such as width, length, length-to-width ratio, and number of striae per 10 µm), several other features observed in the SEM have been suggested as useful for species identification, including (1) the pattern of surface smoothness on the rapheless valve; (2) the number of areolae per stria on the rapheless valve; and (3) whether the striae on the rapheless valve interrupt at the valve margin, thus influencing the number of areolae at the valve edge (Wetzel et al. 2019). In addition to morphological studies, molecular tools have provided new insights into the taxonomy and phylogeny of *Planothidium*. Molecular research has confirmed the presence of two distinct clades within the genus, one with species possessing a sinus or cavum in the rapheless valve and the other lacking these structures (Jahn et al. 2017).

Currently, research on the genus *Planothidium* in China remains limited. In marine environments, one new species has been reported and described (Li et al. 2024). However, in freshwater ecosystems, only a few species have been recorded as new records, and no new species have been reported so far (Liu et al. 2015, 2016; Wang et al. 2019). Karst ecosystems, characterized by carbonate-rich and nutrient-poor waters, remain underexplored. These unique habitats may drive morphological differentiation and adaptive evolution, offering immense potential for diatom taxonomy studies. Recently, several new diatom species have been described from karst regions, such as *Sellaphoragologonica* Lai, Ector & Wetzel, *Germainiellalegionensis* Blanco, Borrego-Ramos & Olenici, *Achnanthidiummediolanceolatum* Yu, You & Kociolek, *Amphorabaotuensis* Li, Nagumo & Xu, and *Fallaciacinariana* Sömek, Hamilton, Solak, Beauger & Sevindik et al. The discovery of these species highlights the underexplored diatom biodiversity in karst environments (Borrego-Ramos et al. 2018; Lai et al. 2018; You et al. 2019b; Li et al. 2022; Sömek et al. 2025).

In recent years, we carried extensive surveys of diatoms species diversity of China (Kociolek et al. 2016b, 2016a, 2019; Lowe et al. 2017; You et al. 2017, 2019a; Yu et al. 2017; Zhou et al. 2024). Based on the collections developed for these studies, four new *Planothidium* species within karst landform of Guizhou have been discovered. This study provides detailed descriptions, as well as light and scanning electron microscopy of these new species: *Planothidiumpseudoreichardtii* sp. nov., *Planothidiumliboensis* sp. nov., *Planothidiumangustirostratum* sp. nov. and *Planothidiummaolanensis* sp. nov., comparing them with similar species within the genus. This work contributes valuable reference material for the taxonomy and morphology of freshwater *Planothidium*, while emphasizing the importance of karst habitats as biodiversity hotspots for diatoms.

## ﻿Materials and methods

Diatom samples were collected from Xiaoqikong Scenic Area and Maolan Nature Reserve (Libo County, Guizhou Province), which is characterized by a subtropical climate and is a typical karst landscape. The sampling sites were distributed in various water bodies, including streams, ponds and underground rivers. The water parameters (pH, Temperature and Conductivity) were measured using a YSI Pro Plus multiparameter meter (YSI, Ohio, USA). Samples were collected using tweezers and a knife, then placed into sealed plastic bottles with the addition of formalin for preservation (final concentration of 4%). Information about the sampling sites is listed in Table 1.

**Table 1.** Locality data and habitat for samples studied.

No. of samples	Location	Coordinates	Habitat	Altitude (m)	Water Temp (°C)	pH	Cond. (μS/cm)	Collector	Collection Date
GZ201510041P	Xiaoqikong Scenic Area	25°15'41"N, 107°45'19"E	Attached to rocks in the pond	780	18.0	7.5	226	Kociolek & Wang Q.X.	10.2.2015
GZ201510045	Xiaoqikong Scenic Area	25°15'36"N, 107°45'16"E	Attached to the stones in the rushing water	780	18.0	7.5	226	Wang Q.X. & Kociolek J.P.	10.2.2015
GZ201510051	Xiaoqikong Scenic Area	25°15'36"N, 107°45'16"E	Attached to floating things in the pond	780	18.0	7.5	226	Wang Q.X. & Kociolek J.P.	10.2.2015
GZ201510066	Xiaoqikong Scenic Area	25°15'02"N, 107°42'46"E	Attached to rocks beneath the waterfall	629	19.5	8.0	215	Wang Q.X. & Kociolek J.P.	10.2.2015
GZ201510099	Maolan Nature Reserve	25°17'32"N, 108°04'16"E	Floating in the slow subsurface stream	650	18.0	7.9	203	Wang Q.X. & Kociolek J.P.	10.4.2015
GZ201510100	Maolan Nature Reserve	25°17'32"N, 108°04'16"E	Floating in the slow subsurface stream	650	18.0	7.9	203	Wang Q.X. & Kociolek J.P.	10.4.2015
GZ201510108	Maolan Nature Reserve	25°17'35"N, 108°04'42"E	Attached to rocks in a rapid river.	811	18.0	7.8	205	Wang Q.X. & Kociolek J.P.	10.4.2015

In the laboratory, samples were processed to remove organic matter and excess impurities using a microwave-assisted reaction system (Model MARS, CEM Corporation, Charlotte, USA) with concentrated nitric acid. The specific steps for sample processing and methods of preservation are as described in You et al. (2021). Clean samples after processing were prepared for both light microscopy (LM) and scanning electron microscopy (SEM). For LM, samples were encapsulated in Naphrax and observed using an Olympus BX-53 microscope with DIC optics and a 1.4 numerical aperture, 100× oil immersion objective. For SEM, the same samples were dried on metal stubs and imaged using a SU8010 SEM at 2 kV with a working distance (WD) of less than 6 mm (Hitachi High-Technologies Corp., Tokyo, Japan). To determine the relative abundance of the new species and record co-occurring diatom species, 400 intact and identifiable diatom valves from each sample were identified under light microscopy (1000×). The images were compiled using Adobe Photoshop 2023. The morphological terminology follows (Morales 2006; Van De Vijver et al. 2018; Wetzel et al. 2019; Morais et al. 2020). The holotype images of each species correspond to specimens that have been circled on the permanent slides. All samples and permanent slides are stored at Lab of Algae and Environment, College of Life Sciences, Shanghai Normal University.

## ﻿Results

### ﻿Phylum Bacillariophyta

**Class Bacillariophyceae Haeckel, 1878**

**Order Achnanthales Silva, 1962**

**Family Achnanthidiaceae Mann, 1990**

**Genus *Planothidium* Round & Bukhtiyarova, 1996**

#### ﻿ Planothidium pseudoreichardtii

Taxon classificationPlantaeAchnanthalesAchnanthidiaceae

Q-M. You, P. Yu & J.P. Kociolek sp. nov.

09DF36F8-ECAA-562C-8006-2B02ACA36692

Figs 1 , 2

##### Holotype.

**Figure 1.**
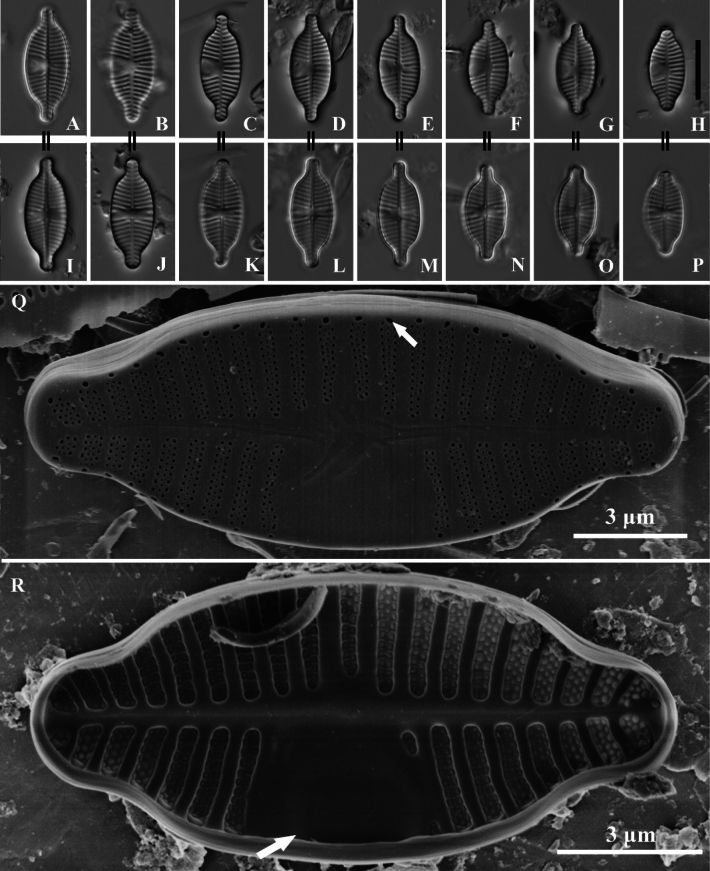
*Planothidiumpseudoreichardtii* sp. nov. LM; **A–H.** Rapheless valves; **I–P.** Raphe valves. “=” indicates the different valves of the same frustule. **Q.**SEM external view of an entire rapheless valve. The white arrow points to a larger areola. **R.**SEM internal view of an entire rapheless valve, showing a distinct sinus in the central area (white arrow). Scale bar as shown. Scale bars: 10 µm (**A–P**).

SHTU! Slide GZ201510045, holotype illustrated in Fig. 1C, J. Diatom samples are housed in the Lab of Algae and Environment, College of Life Sciences, Shanghai Normal University, China.

##### Isotype.

COLO! Material 11111, Slides are housed in the Kociolek Collection, University of Colorado, Museum of Natural History Diatom Herbarium, Boulder, U.S.A.

##### Type locality.

China. Xiaoqikong Scenic Area, Libo County, Guizhou Province, 25°15'36"N, 107°45'16"E, altitude: 780 m, collected by Kociolek J.P. & Wang Q.X., on October 2, 2015.

##### Description.

Light microscopy (LM) (Fig. 1A–P). Valves elliptical with slightly constricted ends and rostrate extensions. Valve dimensions (n = 60): Length 11.5–19.0 µm, width 5.5–7.5 µm. Rapheless Valve (Fig. 1A–H): Axial area narrow, straight, linear. Central area features a U-shaped hyaline region widening towards the valve margin, on the opposite side, striae extend to the axial area. Striae radiate along the valve outline, 14–17 in 10 µm (measured opposite hyaline area). Raphe Valve (Fig. 1I–P): Axial area narrow, straight, linear. Central area rectangular to nearly circular, bordered by 3–4 shortened, asymmetrical striae on each side. Raphe branches straight. Striae radiate along the valve outline, 14–18 in 10 µm.

**Figure 2.**
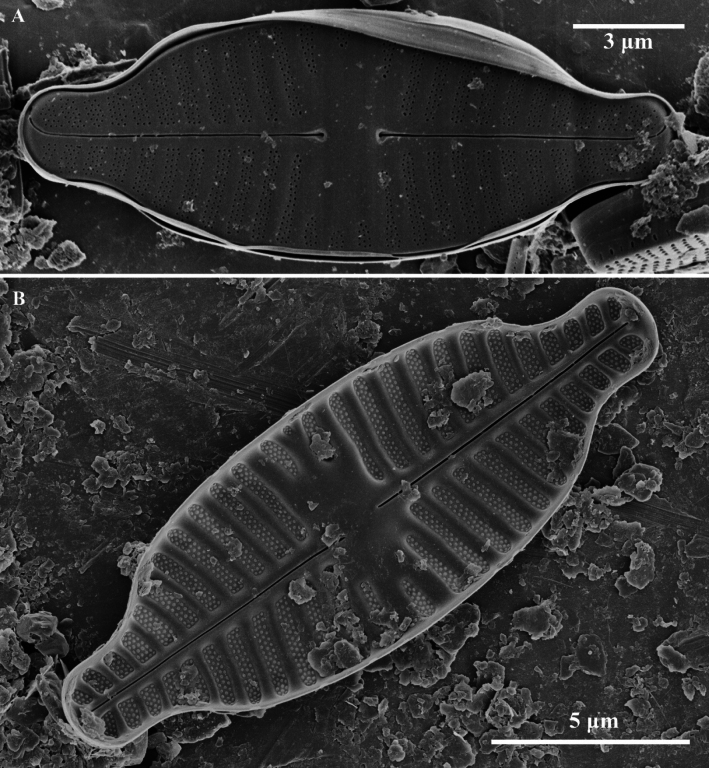
*Planothidiumpseudoreichardtii* sp. nov. SEM; **A.** External view of an entire raphe valve. **B.** Internal view of an entire raphe valve, scale bar as shown. Scale bar as shown.

Scanning electron microscopy (SEM) (Figs 1Q, R, 2A, B). Rapheless Valve (Fig. 1Q, R): Striae composed of 3–4 rows of circular areolae, the outer two rows larger than those near the center. Striae narrow to 2 rows towards the axial area and may expand to 3–4 rows near the valve margin, a larger areola marks the end of striae near the valve margin (Fig. 1Q, white arrow). The central and axial areas feature irregular, shallow, linear depression. Internally, areolae are covered. A clearly visible sinus is present on one side of the central area, forming a shallow circular depression (Fig. 1R, white arrow). Raphe Valve (Fig. 2A, B): Striae composed of 3–4 rows of circular areolae. Striae narrow towards one end near the axial area. The central area typically displays 2 to 3 shortened striae. Striae almost extend onto the valve margin. Raphe branches are straight, with proximal raphe endings expanding into pores, surrounded by shallow, drop-like depressions. Distal raphe ends are curved in the same direction, briefly extending onto the mantle. Internally, proximal raphe endings are slightly deflected to the opposite side, distal raphe endings terminate in a small helictoglossa. Central nodule is raised, with striae wider raised virgae and sunken between them, and areolae are individually covered.

##### Etymology.

The species was named for its outline being similar to *P.reichardtii*.

##### PhycoBank registration.

http://phycobank.org/105527

##### Distribution and ecology.

This species has currently only been found at its type locality, where it was collected from rocks in a rapid stream (water temperature 18.0 °C, elevation 780 m). The species was observed in sample GZ201510041P (2.0%), GZ201510045 (2.5%). In the type material (GZ201510045), *P.pseudoreichardtii* was rare. The associated diatom flora included *Naviculalundii* Reichardt (19.5%), *Achnanthidiumdelmontii* Pérès, le Cohu & Barthès (12.0%), *Planothidiumrostratum* (Østrup) Lange-Bertalot (6.0%), *Platessahustedtii* Lange-Bertalot (5.5%), and *Achnanthidiumminutissimum* (Kützing) Czarnecki (5.0%), and other taxa with lower abundance (less than 5%) such as *Naviculacapitatoradiata* Germain, *Achnanthidiumrivulare* Potapova & Ponader, *Staurosiraconstruens* Ehrenberg, *Punctastriatanyingchiensis* Luo & Wang, *Cocconeisplacentula* Ehrenberg, and *Nitzschiapalea* Smith. Additional ecological information is presented in Table 1.

##### Remarks.

Due to their similar valve outlines, *Planothidiumpseudoreichardtii* sp. nov. and *Planothidiumliboensis* sp. nov. were compared with several morphologically similar taxa, including *Planothidiumreichardtii* Lange-Bertalot & Werum, *Planothidiumrostratum* (Østrup) Lange-Bertalot and *Planothidiumxinguense* Morais, Wetzel & Bicudo (Table 2). However, *P.pseudoreichardtii* sp. nov. can be easily distinguished from *P.liboensis* sp. nov. because the former possesses a sinus, while the latter has a cavum on the center of the rapheless valve. Meanwhile, *P.pseudoreichardtii* can be differentiated from *P.reichardtii* by several distinct features: (1) in LM, the central area of the rapheless valve in *P.pseudoreichardtii* has a broader hyaline area outlined by 3–5 striae, whereas in *P.reichardtii*, the hyaline area is outlined by only 1–2 striae; (2) The striae on the rapheless valve of *P.pseudoreichardtii* consist of 3–4 rows of circular areolae, with the outermost two rows being larger than the central ones, whereas *P.reichardtii* has uniformly sized areolae in its striae; (3) the striae of *P.pseudoreichardtii* terminates at the valve margin with a distinctly enlarged areola, while *P.reichardtii* doesn’t have it. (4) irregular shallow slit-like depressions are present in the central and axial areas of the rapheless valve in *P.pseudoreichardtii*, whereas no depressions were observed on the rapheless valve of *P.reichardtii*.

**Table 2.** Comparison of morphological characteristics of *Planothidiumpseudoreichardtii* sp. nov. & *Planothidiumliboensis* sp. nov. and closely related taxa.

	*Planothidiumpseudoreichardtii* sp. nov.	*Planothidiumreichardtii* Lange-Bertalot & Werum	*Planothidiumliboensis* sp. nov.	*Planothidiumrostratum* (Østrup) Lange-Bertalot	*Planothidiumxinguense* Morais
Reference	This study	Werum and Lange-Bertalot 2004	This study	Lange-Bertalot 1999; Wetzel et al. 2019	Morais et al. 2020
Valve outline	Elliptical	Elliptical to linear-elliptical	Elliptical	Elliptical	Elliptical, asymmetrical
Apices	Rostrate	Subrostrate to capitate	Rostrate	Short to long rostrate	Short to long rostrate
Length	11.5–19.0 µm	8–18 µm	12–18 µm	6.5–15.0 µm	12–14 µm
Width	5.5–7.5 µm	4.0–6.5 µm	5–7 µm	4.0–6.5 µm	6.7–8.2 µm
Central area (RV)	Rectangular to nearly circular	Nd	Rectangular to nearly circular	Rectangular to slightly round	Irregular, rectangular to slightly rounded
Axial area (RV)	Narrow linear	linear	Narrow linear	Narrow, linear, widening towards the central area	Narrow linear
Striae (RV)	14–18/10 µm	Nd	14–17/10 µm	12–16/10 µm	12–14 µm
Areolae composition of striae (RV)	3–4 rows areolae	Nd	4–5 rows areolae	3–4 rows areolae	3–4 rows areolae
Axial area (SV)	Narrow, linear to lanceolate	linear	Narrow linear	Narrow, straight, linear	Narrow, linear, expanded in the central area opposite to the cavum
Additional structure (SV)	Sinus	Sinus	Cavum	Cavum	Cavum
Striae (SV)	14–17/10 µm	Nd	15–16/10 µm	12–14/10 µm	12–14 µm
Areolae composition of striae (SV)	3–4 rows areolae	Nd	4–5 rows areolae	3–4 rows areolae	1–3 rows areolae

Note: “Nd” indicates no data available in the reference.

#### ﻿ Planothidium liboensis

Taxon classificationPlantaeAchnanthalesAchnanthidiaceae

Q-M. You, P. Yu & J.P. Kociolek sp. nov.

233C8D09-6BE2-5097-A314-A5F236BF6C22

Figs 3 , 4

##### Holotype.

**Figure 3.**
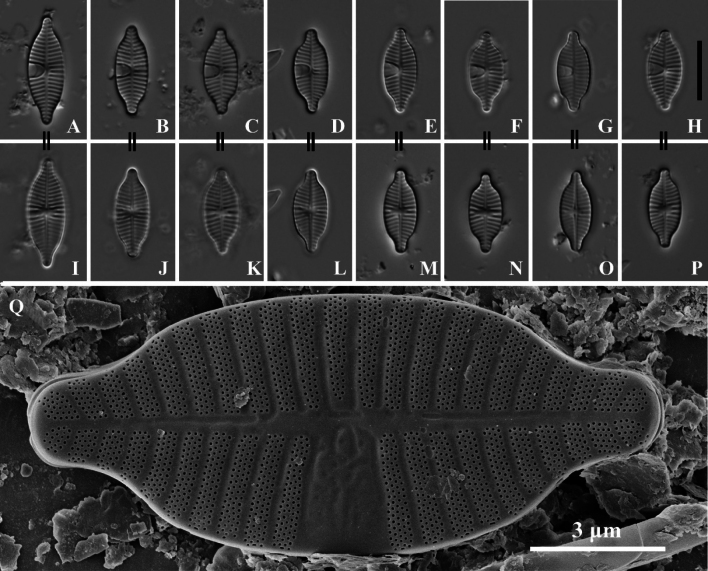
*Planothidiumliboensis* sp. nov. LM; **A–H.** Rapheless valves; **I–P.** Raphe valves. “=” indicates the different valves of the same frustule. **Q.**SEM external view of an entire rapheless valve, scale bar as shown. Scale bars: 10 µm (**A–P**).

SHTU! Slide GZ201510051, holotype illustrated in Fig. 3F, N. Diatom samples are housed in the Lab of Algae and Environment, College of Life Sciences, Shanghai Normal University, China.

##### Isotype.

COLO! Material 11117, Slides are housed in the Kociolek Collection, University of Colorado, Museum of Natural History Diatom Herbarium, Boulder, U.S.A.

##### Type locality.

China. Xiaoqikong Scenic Area, Libo County, Guizhou Province, 25°15'36"N, 107°45'16"E, altitude: 780 m, collected by Wang Q.X. & Kociolek J.P., on October 2, 2015.

##### Description.

Light microscopy (LM) (Fig. 3A–P). Valves elliptical with slightly constricted ends and rostrate extensions. Valve dimensions (n = 75): Length 12–18 µm, width 5–7 µm. Rapheless Valve (SV) (Fig. 3A–H): Axial area narrow, straight, linear. Central area with a large, unilateral, horseshoe-shaped hyaline area containing a clearly visible cavum, on the opposite side, striae extend to the axial area. Striae radiate along the valve outline, 15–16 in 10 µm (measured opposite the cavum). Raphe Valve (RV) (Fig. 3I–P): Axial area narrow, straight, and linear, widening slightly towards the central area. Central area irregular, rectangular to slightly rounded, bordered by 2–3 shortened, asymmetrical striae on each side. Raphe branches straight, with proximal raphe endings drop-like. Striae radiate along the valve outline, 14–17 in 10 µm.

**Figure 4.**
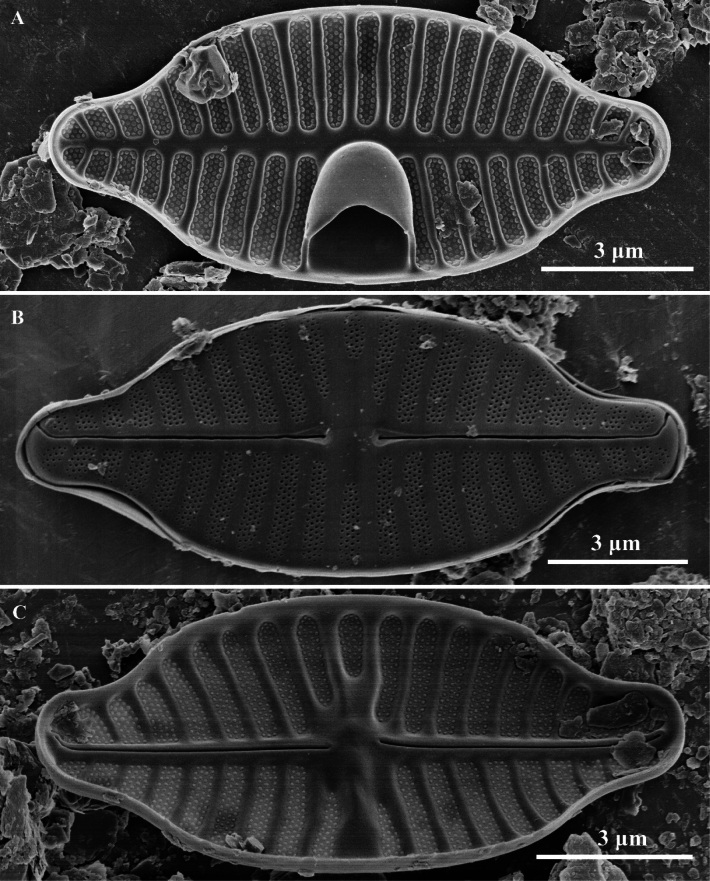
*Planothidiumliboensis* sp. nov SEM; **A.** Internal view of an entire rapheless valve; **B.** External view of an entire raphe valve; **C.** Internal view of an entire raphe valve. Scale bar as shown.

Scanning electron microscopy (SEM) (Figs 3Q, 4A–C). Rapheless Valve (Figs 3Q, 4A): Striae composed of 4 to 5 rows of circular areolae, typically parallel to each other. Near the central area, striae often narrow to 3 areolae adjacent to the axial area. Striae extend onto the valve margin, and no areolae exist on the mantle. The axial area and central area exhibit irregular depressions. The cavum opening is broad, striae are distinctly wider than the virgae and sunken between them, areolae are covered by individual hymenes. Raphe Valve (Fig. 4B, C): Striae composed of 4 to 5 rows of circular areolae, with some striae consisting of 3 areolae near the axial area and valve margin. Striae extend onto the valve margin, and no areolae exist on the mantle. The central area typically displays 2 to 3 shortened areolae. Raphe branches are straight, with proximal raphe endings expanding into pores, surrounded by shallow, drop-like depressions. Distal raphe ends are curved in the same direction, briefly extending onto the mantle. Internally, proximal raphe endings are slightly deflected to the opposite side, distal raphe endings terminate in a small helictoglossa. The central nodule is raised, striae are markedly wider than the virgae and sunken between them, and areolae are covered by individual hymenes.

##### Etymology.

The species was named for the type locality, Libo County.

##### PhycoBank registration.

http://phycobank.org/105528

##### Distribution and ecology.

This species has currently only been found at its type locality, where it was collected from rocks and floating debris in ponds, as well as from rocks beneath a waterfall (water temperature 18.0–19.5 °C, elevation 629–780 m). The species was observed in samples GZ201510045 (1.0%), GZ201510051 (5.8%), and GZ201510066 (1.5%). In the type material (GZ201510051), *P.liboensis* exhibited a relatively high abundance. The associated diatom flora included *Sellaphora* sp. (15.5%), *Planothidiumrostratum* (12.5%), *Fallacia* sp. (5.0%), *Achnanthidiumminutissimum* (5.0%), *Nitzschiapalea* (5.0%), and other taxa with lower abundance (less than 5%) such as *Planothidiumellipticum* (Cleve) Round & Bukhtiyarova, *Sellaphorarotunda* Wetzel, Ector, Van de Vijver, Compère & Mann, *Achnanthidium* spp., *Punctastriatanyingchiensis*, *Staurosiraconstruens*, *Cocconeisplacentula*, and *Platessahustedtii*. Additional ecological information is presented in Table 1.

##### Remarks.

In LM, *P.liboensis*, *P.rostratum*, and *P.xinguense* exhibit broadly similar valve outlines, and all display a cavum. However, *P.liboensis* can be distinguished from other taxa by the following features: (1) *P.liboensis* has a denser arrangement of striae, typically consisting of 4–5 rows of similarly-sized round areolae, and the striae are distinctly wider than the virgae. In contrast, the striae of *P.rostratum* in the rapheless valve consist of 3–4 rows of areolae, where the two middle rows are smaller than the outer rows. The striae of *P.xinguense* in rapheless valve are composed of 1–3 rows of areolae; (2) The central area of rapheless valve in *P.liboensis* exhibits distinctive irregularly shaped shallow depressions, whereas *P.rostratum* features shallow, slit-like depressions. *P.xinguense* also exhibits irregularly shaped shallow depressions in the central area, but their shape differs from those in *P.liboensis*. Additionally, *P.xinguense* has slit-like depressions in the axial area; (3) compared to *P.rostratum*, the cavum of *P.liboensis* has a more open aperture; (4) compared to *P.xinguense*, *P.liboensis* exhibits weaker striae radiation and a narrower central area on the rapheless valve.

#### ﻿ Planothidium angustirostratum

Taxon classificationPlantaeAchnanthalesAchnanthidiaceae

Q-M. You, P. Yu & J.P. Kociolek sp. nov.

50A32D80-7886-5F32-AC92-846E8B398F8E

Figs 5 , 6

##### Holotype.

**Figure 5.**
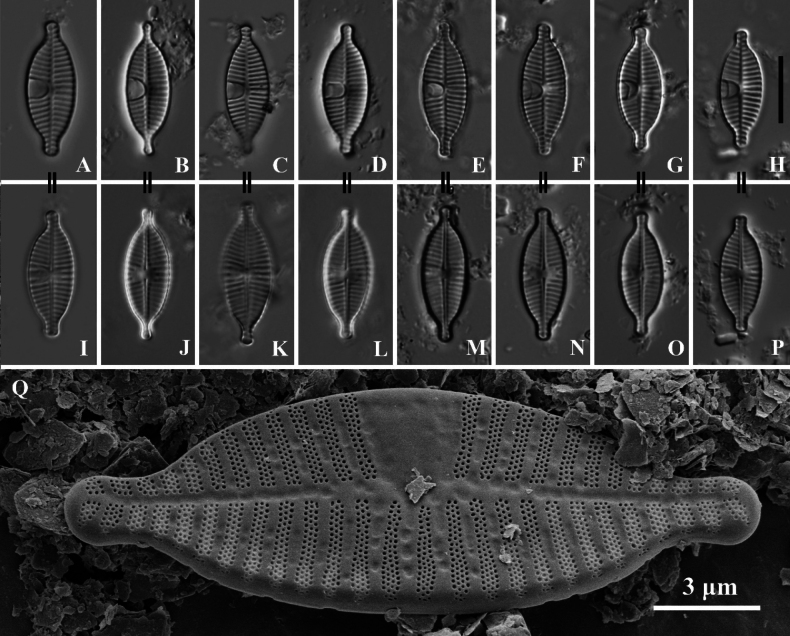
*Planothidiumangustirostratum* sp. nov. LM; **A–H.** Rapheless valves; **I–P.** Raphe valves. “=” indicates the different valves of the same frustule. **Q.**SEM external view of an entire rapheless valve, scale bar as shown. Scale bars: 10 µm (**A–P**).

SHTU! Slide GZ201510051, holotype illustrated in Fig. 5F, N. Diatom samples are housed in the Lab of Algae and Environment, College of Life Sciences, Shanghai Normal University, China.

##### Isotype.

COLO! Material 11117, Slides are housed in the Kociolek Collection, University of Colorado, Museum of Natural History Diatom Herbarium, Boulder, U.S.A.

##### Type locality.

China. Xiaoqikong Scenic Area, Libo County, Guizhou Province, 25°15'36"N, 107°45'16"E, altitude: 780 m, collected by Wang Q.X. & Kociolek J.P., on October 2, 2015.

##### Description.

Light microscopy (LM) (Fig. 5A–P). Valves elliptic-lanceolate with narrowly rostrate to subcapitate apices. Valve dimensions (n = 40): Valve length 17–21 µm, width 6.5–7.0 µm. Rapheless Valve (Fig. 5A–H): Axial area narrow, linear, slightly broadened in the middle. A horseshoe-shaped hyaline area present on one side of the central area, containing a cavum. Striae weakly radiate along the valve outline, 13–14 in 10 µm (measured opposite the hyaline area). Raphe Valve (Fig. 5I–P): Axial area narrow, straight, linear. Central area subcircular to rectangular, bordered by 3–4 slightly shortened striae on each side. Raphe branches straight, with proximal raphe endings drop-like. Striae radiate along the valve outline, 13–14 in 10 µm.

**Figure 6.**
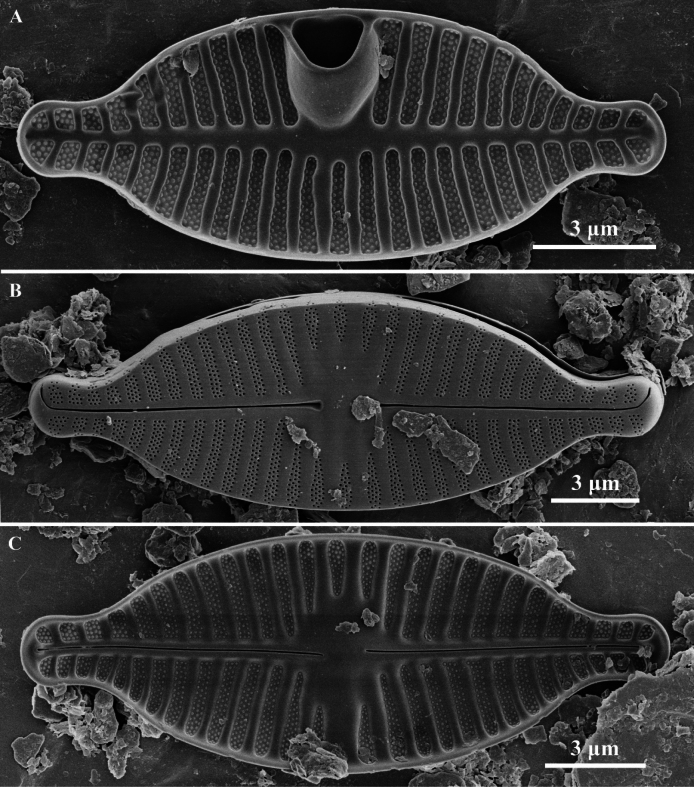
*Planothidiumangustirostratum* sp. nov. SEM; **A.** Internal view of an entire rapheless valve; **B.** External view of an entire raphe valve; **C.** Internal view of an entire raphe valve. Scale bar as shown.

Scanning electron microscopy (SEM) (Figs 5Q, 6A–C). Rapheless Valve (Figs 5Q, 6A): Striae composed of 3–4 rows of circular areolae, narrowing near the central area adjacent to the axial area. Striae extend onto the valve mantle. Irregular circular depressions present between the axial area, central area, even between striae. Internally, areolae covered individually, striae wider than virgae and sunken between them. Cavum opening slightly constricted. Raphe Valve (Fig. 6B, C): Striae composed of 3–4 rows of areolae, striae near the central area regularly shortened and narrowing towards the axial area. Striae extend onto the valve mantle. Raphe branches straight, with proximal raphe endings expanding into pores surrounded by drop-like depressions. Distal raphe ends are curved in the same direction, slightly extending onto the valve mantle. Internally, proximal raphe endings slightly deflected to the opposite side, and distal raphe endings terminating in a faint helictoglossa. Internally, areolae covered individually, striae wider than virgae and sunken between them.

##### Etymology.

The species was named for its narrowly rostrate valve apices.

##### PhycoBank registration.

http://phycobank.org/105529

##### Distribution and ecology.

This species has currently only been found at its type locality, where it was collected from floating debris in a pond (water temperature 18.0 °C, elevation 780 m). The species was observed only in sample (GZ201510051) at a low relative abundance (0.75%). In the type material, *P.angustirostratum* was rare. The associated diatom flora included *Sellaphora* sp. (15.5%), *Planothidiumrostratum* (12.5%), *Planothidiumliboensis* (5.75%), *Fallacia* sp. (5.0%), *Achnanthidiumminutissimum* (5.0%), *Nitzschiapalea* (5.0%), and other taxa with lower abundance (less than 5%) such as *Planothidiumellipticum*, *Sellaphorarotunda*, *Achnanthidium* spp., *Punctastriatanyingchiensis*, *Staurosiraconstruens*, *Cocconeisplacentula*, and *Platessahustedtii*. Additional ecological information is presented in Table 1.

##### Remarks.

Due to the broadly similar valve outline in LM and the presence of a cavum, *Planothidiumangustirostratum* sp. nov. can be compared with *Planothidiumbrasiliense* Wetzel & Blanco, *Planothidiumpotapovae* Wetzel & Ector and *Planothidiumrostratoholarcticum* Lange-Bertalot & Bąk (Table 3). In addition to differences in striae density and valve length and width, *Planothidiumangustirostratum* sp. nov. also can be distinguished from these similar species by the following features: (1) with narrower rostrate to subcapitate apices and a narrower cavum aperture; (2) a smaller central area, ranging from rectangular to nearly circular; (3) its striae denser on the raphe valve, consist of 3–4 rows of circular areolae; (4) circular shallow depressions are present in the central area, axial area, and between the striae, unlike the slit-like depressions observed in *P.potapovae* and *P.rostratoholarcticum*.

**Table 3.** Comparison of morphological characteristics of *Planothidiumangustirostratum* sp. nov. and closely related taxa.

	*Planothidiumangustirostratum* sp. nov.	*Planothidiumbrasiliense* Wetzel & Blanco	*Planothidiumpotapovae* Wetzel & Ector	*Planothidiumrostratoholarcticum* Lange-Bertalot & Bak
Reference	This study	Wetzel et al. 2019	Wetzel et al. 2019	Bąk and Lange-Bertalot 2014; Wetzel et al. 2019
Valve outline	Elliptic-lanceolate	Lanceolate, elliptic-lanceolate	Lanceolate to broadly elliptic–lanceolate	Elliptical
Apices	Narrowly rostrate to subcapitate	Rostrate	Strongly rostrate	Subrostrate
Length	17–21 µm	20–28 µm	11.5–14.5 µm	6.4–13.5 µm
Width	6.5–7.0 µm	7.0–8.0 µm	5.0–6.0 µm	3.8–6.4 µm
Central area (RV)	Subcircular to rectangular	Rectangular	Wide rectangular	Irregular
Axial area (RV)	Narrow linear	Narrow linear	Wide rectangular	Narrow, linear
Striae near to central area (RV)	3–4 shortened striae on each side	2–3 shortened striae on each side	1–3 shortened striae on each side	Usually 1 shortened striae on each side
Raphe	Straight	Straight	Straight	Straight
Striae (RV)	13–14/10 µm	Nd	Nd	14–17/10 µm
Areolae composition of striae (RV)	3–4 rows areolae	Nd	4 rows areolae	2–3 rows areolae
Axial area (SV)	Narrow linear	Narrow, linear, expanded in the central area	Linear–lanceolate,widening in the central area*	Narrow, straight, linear
Additional structure (SV)	Cavum	Cavum	Cavum	Cavum
Striae (SV)	13–14/10 µm	15–17/10 µm	12–13/10 µm	13–17/10 µm
Areolae composition of striae (SV)	3–4 rows areolae	Nd	3–4 rows areolae	2–3 rows areolae

Note: “Nd” indicates no data available in the reference.

#### ﻿ Planothidium maolanensis

Taxon classificationPlantaeAchnanthalesAchnanthidiaceae

Q-M. You, P. Yu & J.P. Kociolek sp. nov.

C92B2E99-9C1F-5D70-A6AC-6EF828CC24C1

Figs 7 , 8

##### Holotype.

**Figure 7.**
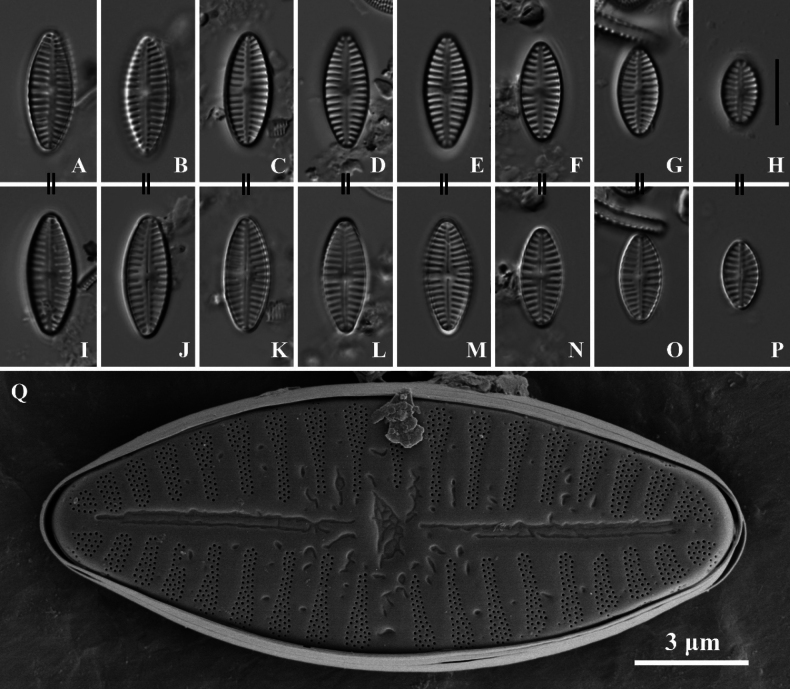
*Planothidiummaolanensis* sp. nov. LM; **A–H.** Rapheless valves; **I–P.** Raphe valves. “=” indicates the different valves of the same frustule. **Q.**SEM external view of an entire rapheless valve, scale bar as shown. Scale bars: 10 µm (**A–P**).

SHTU! Slide GZ201510099, holotype illustrated in Fig. 7D, L. Diatom samples are housed in the Lab of Algae and Environment, College of Life Sciences, Shanghai Normal University, China.

##### Isotype.

COLO! Material 11165, Slides are housed in the Kociolek Collection, University of Colorado, Museum of Natural History Diatom Herbarium, Boulder, U.S.A.

##### Type locality.

China. Maolan Nature Reserve, Libo County, Guizhou Province, 25°17'32"N, 108°04'16"E, altitude: 650 m, collected by Kociolek J.P. & Wang Q.X., on October 4, 2015.

##### Description.

Light microscopy (LM) (Fig. 7A–P). Valves elliptical, with rounded ends not extended. Valve dimensions (n = 50): Length 9.5–18.0 µm, width 5.5–7.5 µm. Rapheless Valve (Fig. 7A–H): Axial area linear-lanceolate, with central area slightly broadened. One side of the central area has two striae with widened spacing, opposite side striae slightly shortened. Striae weakly radiate along the valve outline, narrowing towards axial area, 10–12 in 10 µm (measured opposite hyaline area). Raphe Valve (Fig. 7I–P): Axial area linear-lanceolate, slightly widened in the central area. Central area subcircular, bordered by 2–3 slightly shortened striae on each side. One side of the central area has two striae with widened spacing. Striae radiate weakly, 10–12 in 10 µm. Raphe branches straight, occasionally slightly curved.

**Figure 8.**
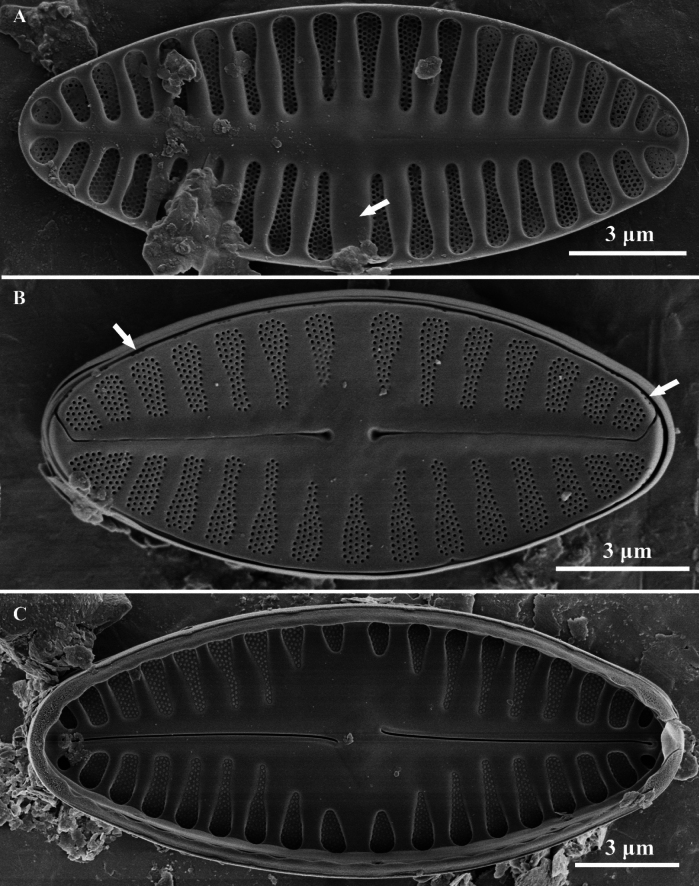
*Planothidiummaolanensis* sp. nov SEM; **A.** Internal view of an entire rapheless valve. The white arrow points to a distinctly wider virgae; **B.** External view of an entire raphe valve. White arrows indicate the areolae on the mantle. **C.** Internal view of an entire raphe valve. Scale bar as shown.

Scanning electron microscopy (SEM) (Figs 7Q, 8A–C). Rapheless Valve (Figs 7Q, 8A): Striae composed of 4–5 rows of circular areolae, markedly narrow near the axial area, reducing to 2–3 rows of areolae, striae almost extend onto the valve margin, and some areolae exist on the mantle. Irregular depressions present on the axial area, central area and even on the virgae. Internally, areolae covered. Virgae widen gradually from the ends towards the center, with striae sunken between virgae, a distinctly wider virgae present on one side of the central area (Fig. 8A, White arrow). Raphe Valve (Fig. 8B, C): Striae composed of 5–6 rows of small circular areolae, markedly narrowing near the central area, reducing to 2–3 rows of areolae. striae don’t extend onto the valve margin, and some areolae exist on the mantle (Fig. 8B, White arrows). Raphe branches straight, occasionally slightly curved. Externally, proximal raphe endings expand into pores, distal raphe ends are curved in the same direction, and extend onto the mantle. Internally, proximal raphe endings slightly deflected to the opposite side, and distal raphe endings terminate in a small helictoglossa. Internally, areolae covered individually by membranes, striae sunken between virgae.

##### Etymology.

The species was named for the Maolan Nature Reserve, where the type specimen was collected.

##### PhycoBank registration.

http://phycobank.org/105530

##### Distribution and ecology.

This species has currently only been found at its type locality, where it was collected from rocks in a subsurface stream and in rapidly flowing river water (water temperature 18.0 °C, elevation 650–811 m). The species was observed in samples GZ201510099 (3.75%), GZ201510100 (0.25%), and GZ2015108 (0.75%). In the type material (GZ201510099), *P.maolanensis* was rare. The associated diatom flora included *Cocconeisplacentula* (23.0%), *Achnanthidiumdelmontii* (7.5%), *Naviculalundii* (7.5%), *Naviculaantonii* Lange-Bertalot, *Navicula* sp. (6.0%), *Achnanthidiumminutissimum* (5.5%), and other taxa with lower abundance (less than 5%) such as *Platessahustedtii*, *Achnanthidium* spp., *Encyonemahophense* Krammer, and *Achnanthesprominula* Levkov & Tofilovska. Additional ecological information is presented in Table 1.

##### Remarks.

Due to the broadly similar valve outline in LM and the absence of cavum, *Planothidiummaolanensis* sp. nov. can be compared with *P.hauckianum* (Grunow) Bukhtiyarova, *P.kaetherobertianum* Van de Vijber & Bosak, and *P.iberense* Rovira & Witkowski (Table 4). *P.maolanensis* can be easily distinguished from *P.kaetherobertianum* and *P.iberense* by valve outline and striae density, while it shows greater overall similarity to *P.hauckianum*. However, *P.maolanensis* can still be differentiated by the following features: (1) In *P.maolanensis*, only two striae on one side of the central area show slightly increased spacing, while the remaining striae are evenly arranged. In contrast, *P.hauckianum* has more striae with increased spacing on both sides of the central area. (2) The striae of *P.maolanensis* consist of 4–5 rows of circular areolae on rapheless valve and 5–6 rows on raphe valve. In *P.hauckianum*, the striae consist of 3–4 rows of circular areolae on the rapheless valve and 2–4 rows on the raphe valve. (3) Although the striae in *P.maolanensis* and *P.hauckianum* both generally narrow toward the axial area, the narrowing in *P.maolanensis* is not gradual or consistent. Sometimes, the middle of striae contracts, making it narrower than the ends near the axial area. (4) *P.maolanensis* has irregularly shaped shallow depressions in axial area and between the striae, while *P.hauckianum* exhibits slit-like depressions.

**Table 4.** Comparison of morphological characteristics of *Planothidiummaolanensis* sp. nov. and closely related taxa.

	*Planothidiummaolanensis* sp. nov.	*Planothidiumhauckianum* (Grunow) Bukhtiyarova	*Planothidiumkaetherobertianum* Van de Vijber & Bosak	*Planothidiumiberense* Rovira & Witkowski
Reference	This study	Kulaš et al. 2020	Van De Vijver and Bosak 2019	Rovira et al. 2011
Valve outline	Elliptical	Linear-lanceolate or elliptic-lanceolate	lanceolate to elliptic-lanceolate in smaller valves with cuneately rounded	Elliptical-lanceolate
Apices	Rounded ends not extended	Very weakly protracted, slightly rostrate to broadly rounded in smaller valves	Only very weakly protracted apices	Moderately produced, obtusely rounded
Length	9.5–18.0 µm	7–30 µm	7–13 µm	17–26 µm
Width	5.5–7.5 µm	4.5–7.0 µm	4.0–4.5 µm	6.5–9.5 µm
Central area (RV)	Subcircular	Asymmetrical	Rounded to rectangular	Circular
Axial area (RV)	Narrow linear	Narrow, linear	Very narrow, linear	Narrow, linear
Raphe	Straight to slightly curved	Straight to weakly curved	Straight	Straight
Striae (RV)	10–12/10 µm	8–10/10 µm	16–17/10 µm	12–15/10 µm
Areolae composition of striae (RV)	5–6 rows areolae	2–4 rows areolae	2–3 rows areolae	4 rows areolae
Axial area (SV)	Narrow linear	Narrow linear	Linear to narrowly lanceolate	Narrow, linear
Additional structure (SV)	Absent	Absent	Absent	Absent
Striae (SV)	10–12/10 µm	7–11/10 µm	15–16/10 µm	14–16/10 µm
Areolae composition of striae (SV)	4–5 rows areolae	3–4 rows areolae	2 rows areolae	3–4 rows areolae

## ﻿Discussion

The four new species described in this study exhibit multiseriate striae on both the raphe valve and the rapheless valve. On one side of the central area of the rapheless valve, *Planothidiumpseudoreichardtii* sp. nov. features an uncovered depression known as a “sinus”, *Planothidiumliboensis* sp. nov. and *Planothidiumangustirostratum* sp. nov. possess a cavum, and *Planothidiummaolanensis* sp. nov. lacks both sinus and cavum. The four new species were compared with several related and morphologically similar taxa (Tables 2, 3, 4).

As a complement to morphometry, Wetzel et al. (2019) proposed several distinguishing features in SEM, such as the pattern of valve surface smoothness on the rapheless valve, the number of areolae per stria, and whether the striae on the rapheless valve are interrupted towards the valve mantle. During the observation of these new *Planothidium* species, we found that the morphology of the striae and the number of areolae rows per stria on the rapheless valve are relatively stable within the same species. For instance, the rapheless valve striae of *P.pseudoreichardtii* sp. nov. are composed of two outer rows of larger areolae and 1–2 smaller rows in the center, with an enlarged areola present at the end of each stria. The presence or absence of striae on the rapheless valve mantle is also stable within the same species. Striae are present on the valve mantle of *P.angustirostratum* sp. nov. and *P.maolanensis* sp. nov., whereas no striae are observed on the valve mantle of *P.liboensis* sp. nov. and *P.maolanensis* sp. nov. Furthermore, we observed that the feature of valve surface smoothness on the rapheless valve is consistent within each species. Based on the shape of the depressions, these features can be roughly classified into three types: *P.angustirostratum* sp. nov. exhibits circular shallow depressions on the valve surface; *P.liboensis* sp. nov. and *P.maolanensis* sp. nov. display irregularly shaped depressions; while *P.pseudoreichardtii* sp. nov. and *P.rostratum*, also observed in the Xiaoqikong Scenic Area, exhibit slit-like shallow depressions.

Currently, numerous species of the genus *Planothidium* have been discovered in various aquatic environments and habitats worldwide (Blanco et al. 2013; N’Guessan et al. 2014; Wetzel and Ector 2014a, 2014b; Kopalová et al. 2016; Riaux-Gobin et al. 2018; Stancheva 2019; Cantonati et al. 2021; Lai et al. 2021). Additionally, some studies have investigated the ecological distribution and physiological characteristics of *Planothidium* species (Sbihi et al. 2014; Stancheva et al. 2020; Juchem et al. 2023). Despite significant advancements over the years, the taxonomy of *Planothidium* is relatively well-developed, but several questions remain open for further exploration. Future research may require additional populations or increased taxa sampling to examine the stability of certain morphological features within the genus. Furthermore, integrating more ecological data and molecular techniques could provide deeper insights into the variability of these features under different environmental conditions and their taxonomic applicability.

## Supplementary Material

XML Treatment for Planothidium pseudoreichardtii

XML Treatment for Planothidium liboensis

XML Treatment for Planothidium angustirostratum

XML Treatment for Planothidium maolanensis

## ﻿Additional information

### Author ORCIDs

Yun Li https://orcid.org/0009-0005-1251-7941

Lin-Xin Lu https://orcid.org/0009-0004-5919-2083

Hui-Wen Zhou https://orcid.org/0009-0004-3926-4887

Pan Yu https://orcid.org/0000-0001-7937-2381

John Patrick Kociolek https://orcid.org/0000-0001-9824-7164

Wan-Ting Pang https://orcid.org/0000-0002-4231-4046

Quan-Xi Wang https://orcid.org/0000-0002-0317-6613

Qing-Min You https://orcid.org/0000-0002-5538-8503

### Data availability

All of the data that support the findings of this study are available in the main text.

## References

[B1] Álvarez-Blanco I Blanco S (2013) *Planothidiumgalaicum* sp. nov. (Bacillariophyta, Achnanthidiaceae), a new diatom species from Galician coast, Spain. Phytotaxa 151: 44. 10.11646/phytotaxa.151.1.4

[B2] Bąk M Lange-Bertalot H (2014) Four small-celled *Planothidium* Species from Central Europe proposed as new to science. Oceanological and Hydrobiological Studies 43: 346–359. 10.2478/s13545-014-0152-9

[B3] Blanco S Álvarez‐Blanco I Cejudo‐Figueiras C Espejo JMR Barrera CB Bécares E Del Olmo FD Artigas RC (2013) The diatom flora in temporary ponds of Doñana National Park (southwest Spain): Five new taxa. Nordic Journal of Botany 31: 489–499. 10.1111/j.1756-1051.2013.01691.x

[B4] Borrego-Ramos M Blanco S Olenici A (2018) Diatoms from the valporquero cave (Leon, NW Spain), with the description of *Germainiellalegionensis* sp. nov. Journal of Caves and Karst Studies 80: 181–189. 10.4311/2017MB0128

[B5] Cantonati M Bilous O Angeli N Van Wensen L Lange-Bertalot H (2021) Three new diatom species from spring habitats in the Northern Apennines (Emilia-Romagna, Italy). Diversity 13: 549. 10.3390/d13110549

[B6] Guiry MD Guiry GM (2024) AlgaeBase. World-wide electronic publication, National University of Ireland, Galway. https://www.algaebase.org [Searched on 05 December 2024]

[B7] Jahn R Abarca N Gemeinholzer B Mora D Skibbe O Kulikovskiy M Gusev E Kusber W-H Zimmermann J (2017) *Planothidiumlanceolatum* and *Planothidiumfrequentissimum* reinvestigated with molecular methods and morphology: Four new species and the taxonomic importance of the sinus and cavum. Diatom Research 32: 75–107. 10.1080/0269249X.2017.1312548

[B8] Juchem DP Schimani K Holzinger A Permann C Abarca N Skibbe O Zimmermann J Graeve M Karsten U (2023) Lipid degradation and photosynthetic traits after prolonged darkness in four Antarctic benthic diatoms, including the newly described species *Planothidiumwetzelii* sp. nov. Frontiers in Microbiology 14: 1241826. 10.3389/fmicb.2023.1241826 PMC10500929 37720158

[B9] Kociolek JP You QM Stepanek J Lowe RL Wang QX (2016a) A new *Eunotia* (Bacillariophyta: Eunotiales) species from Karst formations of southern China. Phytotaxa 265: 285. 10.11646/phytotaxa.265.3.10

[B10] Kociolek JP You QM Stepanek JG Lowe RL Wang QX (2016b) New freshwater diatom genus, *Edtheriotia* gen. nov. of the Stephanodiscaceae (Bacillariophyta) from south‐central China. Phycological Research 64: 274–280. 10.1111/pre.12145

[B11] Kociolek JP You QM Lou F Yu P Lowe RL Wang QX (2019) First report and new freshwater species of *Germainiella* (Bacillariophyta) from the Maolan Nature Reserve, Guizhou Province, China. Phytotaxa 393: 35. 10.11646/phytotaxa.393.1.3

[B12] Kopalová K Zidarova R Van De Vijver B (2016) Four new monoraphid diatom species (Bacillariophyta, Achnanthaceae) from the Maritime Antarctic Region. European Journal of Taxonomy 217: 1–19. 10.5852/ejt.2016.217

[B13] Kulaš A Gligora Udovič M Ector L Van De Vijver B (2020) Analysis of the type material of *Achnantheshauckiana* Grunow (Achnanthaceae, Bacillariophyceae). Botany Letters 167: 439–452. 10.1080/23818107.2020.1808527

[B14] Kulikovskiy MS Andreeva SA Gusev ES Kuznetsova IV Annenkova NV (2016) Molecular phylogeny of monoraphid diatoms and raphe significance in evolution and taxonomy. The Biological Bulletin 43: 398–407. 10.1134/S1062359016050046 30226934

[B15] Lai GG Ector L Lugliè A Sechi N Wetzel CE (2018) *Sellaphoragologonica* sp. nov. (Bacillariophyta, Sellaphoraceae), a new diatom species from a Mediterranean karst spring (Sardinia, Italy). Phytotaxa 356: 145. 10.11646/phytotaxa.356.2.4

[B16] Lai GG Ector L Padedda BM Wetzel CE (2021) *Planothidiummarganaiensis* sp. nov. (Bacillariophyta), a new cavum-bearing species from a karst spring in south-western Sardinia (Italy). Phytotaxa 489: 140–154. 10.11646/phytotaxa.489.2.3

[B17] Lange-Bertalot H (1999) Neue Kombinationen von Taxa aus Achnanthes Bory (*sensu lato*). In: Lange-Bertalot H (Ed.) Iconographia Diatomologica, Vol.6: Phytogeography-Diversity-Taxonomy. Koeltz Scientific Books, Königstein, Germany, 276–289.

[B18] Li YH Nagumo T Xu KD (2022) Morphology and molecular phylogeny of *Amphorabaotuensis* sp. nov., a new freshwater benthic diatom from a karst spring in China. Diatom Research 37: 145–153. 10.1080/0269249X.2022.2063390

[B19] Li L Huang YY Nong QZ Lai JX Li YH (2024) *Planothidiumpseudolinkei* sp. nov. (Bacillariophyta), a new marine monoraphid diatom species from the coast of Guangxi, China. PhytoKeys 246: 237–249. 10.3897/phytokeys.246.128068 39280933 PMC11393488

[B20] Liu Y Fan YW Wang QX (2015) Study on the Achnanthoid Diatoms from the Great Xing’an Moutains. Acta Hydrobiologica Sinica 39: 554–563.

[B21] Liu Y Kociolek JP Wang QX Fan YW (2016) Newly recorded of freshwater monoraphid diatom from Hainan Island, China. Acta Hydrobiologica Sinica 40: 1266–1277.

[B22] Lowe R Kociolek J You QM Wang QX Stepanek J (2017) Diversity of the diatom genus *Humidophila* in karst areas of Guizhou, China. Phytotaxa 305: 269–284.

[B23] Morais KS Costa LF Bicudo CEDM Ector L Wetzel CE (2020) A new *Planothidium* species (Achnanthidiaceae, Bacillariophyceae) from Xingu Ria, Amazon River basin, Brazil. Phytotaxa 477: 194–204. 10.11646/phytotaxa.477.2.4

[B24] Morales EA (2006) Small *Planothidium* Round Et Bukhtiyarova (Bacillariophyceae) taxa related to *P.daui* (foged) Lange-Bertalot from the United States. Diatom Research 21: 325–342. 10.1080/0269249X.2006.9705673

[B25] N’Guessan KR Wetzel CE Ector L Coste M Cocquyt C Van De Vijver B Yao SS Ouattara A Kouamelan EP Tison-Rosebery J (2014) *Planothidiumcomperei* sp. nov. (Bacillariophyta), a new diatom species from Ivory Coast. Plant Ecology and Evolution 147: 455–462. 10.5091/plecevo.2014.981

[B26] Novis PM Braidwood J Kilroy C (2012) Small diatoms (Bacillariophyta) in cultures from the Styx River, New Zealand, including descriptions of three new species. Phytotaxa 64: 11. 10.11646/phytotaxa.64.1.3

[B27] Riaux-Gobin C Witkowski A Igersheim A Lobban CS Al-Handal AY Compère P (2018) *Planothidiumjuandenovense* sp. nov. (Bacillariophyta) from Juan de Nova (Scattered Islands, Mozambique Channel) and other tropical environments: A new addition to the *Planothidiumdelicatulum* complex. Fottea 18: 106–119. 10.5507/fot.2017.019

[B28] Round FE Bukhtiyarova L (1996) Four new genera based on Achnanthes (Achnanthidium) together with a re-definition of *Achnanthidium*. Diatom Research 11: 345–361. 10.1080/0269249X.1996.9705389

[B29] Rovira L Witkowski A Trobajo R Ruppel M Ibáñez C (2011) *Planothidiumiberense* sp. nov., a new brackish diatom of the Ebro Estuary, northeast Spain. Diatom Research 26: 99–107. 10.1080/0269249X.2011.587645

[B30] Sbihi K Cherifi O Bertrand M El Gharmali A (2014) Biosorption of metals (Cd, Cu and Zn) by the freshwater diatom *Planothidiumlanceolatum*: A laboratory study. Diatom Research 29: 55–63. 10.1080/0269249X.2013.872193

[B31] Sömek H Hamilton PB Solak CN Beauger A Yilmaz E Sevindik TO (2025) A new diatom (Bacillariophyta) species—*Fallaciacinariana* sp. nov.—from Kaklık Cave in the Western Anatolian Karst Region, Republic of Türkiye. Phytotaxa 694: 173–183. 10.11646/phytotaxa.694.2.5

[B32] Stancheva R (2019) *Planothidiumsheathii*, a new monoraphid diatom species from rivers in California, USA. Phytotaxa 393: 131. 10.11646/phytotaxa.393.2.4

[B33] Stancheva R Kristan NV Kristan III WB Sheath RG (2020) Diatom genus *Planothidium* (Bacillariophyta) from streams and rivers in California, USA: Diversity, distribution and autecology. Phytotaxa 470: 1–30. 10.11646/phytotaxa.470.1.1

[B34] Van De Vijver B Bosak S (2019) *Planothidiumkaetherobertianum*, a new marine diatom (Bacillariophyta) species from the Adriatic Sea. Phytotaxa 425: 105–112. 10.11646/phytotaxa.425.2.5

[B35] Van De Vijver B Wetzel C Kopalová K Zidarova R Ector L (2013) Analysis of the type material of *Achnanthidiumlanceolatum* Brébisson ex Kützing (Bacillariophyta) with the description of two new *Planothidium* species from the Antarctic Region. Fottea 13: 105–117. 10.5507/fot.2013.010

[B36] Van De Vijver B Wetzel CE Ector L (2018) Analysis of the type material of *Planothidiumdelicatulum* (Bacillariophyta) with the description of two new *Planothidium* species from the sub-Antarctic Region. Fottea 18: 200–211. 10.5507/fot.2018.006

[B37] Wang YL Yu P Cao Y Wang QX You QM (2019) New records of species of Achnanthidiaceae (Bacillariophyta) in Ganzi, Sichuan, China. Zhiwu Kexue Xuebao 37: 10–17. 10.11913/PSJ.2095-0837.2019.10010

[B38] Werum M Lange-Bertalot H (2004) Diatoms in Springs from Central Europe and elsewhere under the influence of hydrologeology and anthropogenic impacts. In: Lange-Bertalot H (Ed.) Iconographia Diatomologica 13.A.R.G. Gantner Verlag K.G, Ruggell, 3–417.

[B39] Wetzel CE Ector L (2014a) *Planothidiumlagerheimii* comb. nov. (Bacillariophyta, Achnanthales) a forgotten diatom from South America. Phytotaxa 188: 261. 10.11646/phytotaxa.188.5.3

[B40] Wetzel CE Ector L (2014b) Taxonomy, distribution and autecology of *Planothidiumbagualensis* sp. nov. (Bacillariophyta) a common monoraphid species from southern Brazilian rivers. Phytotaxa 156: 201. 10.11646/phytotaxa.156.4.2

[B41] Wetzel CE Van De Vijver B Blanco S Ector L (2019) On some common and new cavum-bearing *Planothidium* (Bacillariophyta) species from freshwater. Fottea 19: 50–89. 10.5507/fot.2018.016

[B42] You QM Kociolek JP Cai MJ Lowe RL Liu Y Wang QX (2017) Morphology and ultrastructure of *Sellaphoraconstrictum* sp. nov. (Bacillariophyta), a new diatom from southern China. Phytotaxa 327: 261. 10.11646/phytotaxa.327.3.5

[B43] You QM Yu P Kociolek JP Wang YL Luo F Lowe R Wang QX (2019a) A new species of *Achnanthes* (Bacillariophyceae) from a freshwater habitat in a karst landform from south-central China. Phycological Research 67: 303–310. 10.1111/pre.12381

[B44] You QM Cao Y Yu P Kociolek JP Zang LX Wu B Lowe R Wang QX (2019b) Three new subaerial *Achnanthidium* (Bacillariophyta) species from a karst landform in the Guizhou Province, China. Fottea 19: 138–150. 10.5507/fot.2019.005

[B45] You QM Zhao K Wang YL Yu P Kociolek JP Pang WT Wang QX (2021) Four new species of monoraphid diatoms from Western Sichuan Plateau in China. Phytotaxa 479: 257–274. 10.11646/phytotaxa.479.3.3

[B46] Yu P You QM Kociolek JP Lowe R Wang QX (2017) *Nupelamajor* sp. nov., a new diatom species from Maolan Nature Reserve, central-south of China. Phytotaxa 311: 245. 10.11646/phytotaxa.311.3.4

[B47] Zhou HW Yu P Guo LY Kociolek JP Wang QX You QM (2024) *Cocconeiscrisscrossis* sp. nov., a new monoraphid diatom (Bacillariophyta) from southern China. PhytoKeys 242: 39–50. 10.3897/phytokeys.242.123316 38774390 PMC11106566

